# Characterization of Novel StAR (Steroidogenic Acute Regulatory Protein) Mutations Causing Non-Classic Lipoid Adrenal Hyperplasia

**DOI:** 10.1371/journal.pone.0020178

**Published:** 2011-05-27

**Authors:** Christa E. Flück, Amit V. Pandey, Bernhard Dick, Núria Camats, Mónica Fernández-Cancio, María Clemente, Miquel Gussinyé, Antonio Carrascosa, Primus E. Mullis, Laura Audi

**Affiliations:** 1 Pediatric Endocrinology and Diabetology, University Children's Hospital Bern, Bern, Switzerland; 2 Department of Nephrology and Hypertension, Inselspital, University of Bern, Bern, Switzerland; 3 Department of Pediatrics, Institut de Recerca, Hospital Vall d'Hebron, Centre for Biomedical Research on Rare Diseases (CIBERER), Autonomous University, Barcelona, Spain; Karolinska Insitutet, Sweden

## Abstract

**Context:**

Steroidogenic acute regulatory protein (StAR) is crucial for transport of cholesterol to mitochondria where biosynthesis of steroids is initiated. Loss of StAR function causes lipoid congenital adrenal hyperplasia (LCAH).

**Objective:**

StAR gene mutations causing partial loss of function manifest atypical and may be mistaken as familial glucocorticoid deficiency. Only a few mutations have been reported.

**Design:**

To report clinical, biochemical, genetic, protein structure and functional data on two novel StAR mutations, and to compare them with published literature.

**Setting:**

Collaboration between the University Children's Hospital Bern, Switzerland, and the CIBERER, Hospital Vall d'Hebron, Autonomous University, Barcelona, Spain.

**Patients:**

Two subjects of a non-consanguineous Caucasian family were studied. The 46,XX phenotypic normal female was diagnosed with adrenal insufficiency at the age of 10 months, had normal pubertal development and still has no signs of hypergonodatropic hypogonadism at 32 years of age. Her 46,XY brother was born with normal male external genitalia and was diagnosed with adrenal insufficiency at 14 months. Puberty was normal and no signs of hypergonadotropic hypogonadism are present at 29 years of age.

**Results:**

StAR gene analysis revealed two novel compound heterozygote mutations T44HfsX3 and G221S. T44HfsX3 is a loss-of-function StAR mutation. G221S retains partial activity (∼30%) and is therefore responsible for a milder, non-classic phenotype. G221S is located in the cholesterol binding pocket and seems to alter binding/release of cholesterol.

**Conclusions:**

StAR mutations located in the cholesterol binding pocket (V187M, R188C, R192C, G221D/S) seem to cause non-classic lipoid CAH. Accuracy of genotype-phenotype prediction by *in vitro* testing may vary with the assays employed.

## Introduction

Mutations in the *StAR* gene were first described in patients with classic congenital lipoid adrenal hyperplasia (CLAH) in which both the adrenals and the gonads seemed to completely lack steroidogenesis [1]. The underlying pathophysiology of CLAH is explained by the defective transport of cholesterol needed for the biosynthesis of steroids, to the inner mitochondrial membrane, and the cytoplasmic accumulation of lipid droplets which would fatally harm the cells of the steroidogenic tissues over time [2]. So far, patients described as having the recently described non-classic form of lipoid adrenal hyperplasia (NCLAH) presented with adrenal insufficiency in early infancy or later and, while manifesting with hypocortisolism, had inconsistent signs of mineralocorticoid deficiency [3], [4], [5], thus resembling the clinical profile of familial glucocorticoid deficiency (FGD). FGD has been described as a syndrome secondary to ACTH resistance, and inactivating mutations in the gene encoding melanocortin type 2 receptor (*MC2R*) were the first to be associated with FGD [6]. However, only ∼25% of such patients were found to be the carriers of *MC2R* mutations and have been classified as type 1 FGD [7]. A second genetic cause of FGD was identified in 15–20% of patients resulting from defects in the MC2R receptor accessory protein (*MRAP*) (FGD type 2) [7], [8]. In the remaining 55% of patients with a FGD syndrome, no mutations in either *MC2R* or *MRAP* genes were found. Large family association studies delimited a region on chromosome 8q12.1–q21.2 that was associated with the FGD syndrome in several families (FGD type 3) [9]. The *StAR* gene maps to the gene locus 8p11.2 and has been identified only recently as causing non-classic lipoid adrenal hyperplasia in a first series of patients in 5 families [4]. In contrast to CLAH which leads to a complete undervirilization/feminization of the external genitalia in 46,XY subjects at birth, most 46,XY patients with the NCLAH seem to have a normal gonadal function during their fetal life and therefore have normal male genitalia at birth or show only minimal anomalies such as cryptorchidism or hypospadias [3], [4], [5]. However, some patients that were followed to adulthood showed a progressive decline in the gonadal functions over time, as demonstrated by decreasing plasma testosterone, high gonadotropins and abnormal spermiogram [4].

To better understand whether a StAR mutation will lead to the severe form of CLAH or whether it will manifest as NCLAH mimicking a FGD syndrome, further patients need to be studied and followed as they age. On the other hand, patients manifesting with a FGD syndrome who do not have mutations in the genes for *MC2R* and *MRAP* should be genetically investigated for *StAR* mutations.

In this report, we have studied a series of 7 patients (6 families) who had the clinical and biochemical diagnosis of congenital isolated glucocorticoid deficiency, for possible genetic defects. The *MC2R* gene analysis revealed a novel homozygous MC2R mutation (K289fs) in one patient [10]. The *MRAP* gene sequencing showed a normal sequence in all 7 patients. The *StAR* gene analysis revealed two novel mutations (T44HfsX3 and G221S) present in compound heterozygote form in two siblings. Here we are describing the clinical, genetic and functional characteristics of these novel StAR mutations.

## Materials and Methods

### Case Reports

#### Patient 1

A Caucasian phenotypically normal female was the first child of non-consanguineous parents, born in 1978 after an uneventful pregnancy and a normal neonatal period. At 10 months of age, she experienced an episode of dehydration and hypoglycemia accompanied by skin hyperpigmentation: blood glucose 23 mg/dl (1.27 mmol/L), plasma sodium 127 mmol/L, potassium 4.5 mmol/L, urea 75 mg/dL (12.4 mmol/L). Fifteen days later she suffered a similar episode. The cortisol levels did not respond to stimulation with ACTH [250 µg perfused over 4 h, followed by 250 µg IM/12 h for the following 2 days]: baseline 2.3 µg/dL (63.4 nmol/L) followed by 3.7 µg/dL (102.1 nmol/L) at 15 h, 1.9 µg/dL (52.4 nmol/L) at 24 h, 2.1 µg/dL (57.9 nmol/L) at 48 h and 1.4 µg/dL (38.6 nmol/L) at 72 h. The ACTH was not measured and baseline plasma renin activity was 22 ng/mL/h (28.5 nmol/L/h; normal range: 1.1–22.9 ng/mL/h). Karyotype was 46,XX. She was diagnosed with congenital adrenal hyperplasia and was treated with hydrocortisone (18–20 mg/m^2^/d) and fludrocortisone (50 µg/d); doses were adjusted during childhood and puberty according to the growth and the renin activity control. Pubertal development started at 12 years of age, menarche presented at 14 years, total pubertal growth gain was 14 cm and a final height of 143 cm was reached at the age of 16 years (midparental height 147.2 cm). Currently, at the age of 32 years, she is doing fine on hydrocortisone (10 mg/m^2^/d) and fludrocortisone (50 µg/d) replacement treatment, has regular menses and normal serum gonadotropin concentrations at early follicular phase (LH 2.6 IU/L and FSH 4.2 IU/L); the ACTH is un-measurable.

#### Patient 2

The younger brother of patient 1, was born in 1981 after an uneventful pregnancy. He had normal male genitalia and descended testes. At 3 weeks of life, a Synacthen® (250 µg/1.73 m^2^ iv) test showed a normal cortisol response: baseline 2.5 µg/dL (68.9 nmol/L) and peak 19.5 µg/dL (538.0 nmol/L). However, at 14 months of age he presented with hypoglycemic episodes and skin hyperpigmentation. ACTH was elevated [>1250 pg/mL (>275 pmol/L)], cortisol did not respond to ACTH stimulation [basal 3.1 µg/dL (85.5 nmol/L); peak 3.4 µg/dL (93.8 nmol/L)] and plasma renin activity was normal [4.2 ng/mL/h (5.4 nmol/L/h)]. He was treated with hydrocortisone (16–18 mg/m^2^/d) and fludrocortisone (50 µg/d) during childhood and puberty. Puberty started at 11.5 years of age, hydrocortisone dose was adjusted to 10 mg/m^2^/d to improve growth, total pubertal growth gain was 31 cm and he reached a final height of 159.3 cm (midparental height 160.7 cm). At 17 years of age, fludrocortisone therapy was stopped and plasma renin activity remained normal thereafter. Presently, at the age of 29 years, he is fine on only hydrocortisone therapy. Bilateral testicular volume is 25 mL. Serum testosterone is 669 ng/dL (23.1 nmol/L; normal range 262–1600 ng/dL), LH 7.3 IU/L (normal range 0.8–8.0), FSH 7.2 IU/L (normal range 0.7–12.0), inhibin B 138.2 pg/mL (normal range 107–322 pg/mL), androstendione 150 ng/dL (5.2 nM/L; normal range 70–250 ng/dL), DHEA <70 ng/dL (<2.4 nmol/L; normal range 400–800 ng/dL); DHEA-S <15 µg/dL (<384 nmol/L; normal range 60–410 µg/dL); ACTH is 12 pg/mL (2.6 pmol/L) under cortisol replacement therapy.

### *MC2R*, *MRAP* and *StAR* gene analysis

The study was approved by the ethics committee of the Hospital Vall d'Hebron, Barcelona, Spain, where the human studies were performed. Written informed consent of patients and families was obtained for genetic analysis of FGD. Leukocyte genomic DNA was prepared and the genes for *MC2R*, *MRAP* and *StAR* were analyzed, exon by exon, using specific primers and PCR amplification as previously described [1], [2], [11], [12]. The PCR-amplified products were purified and sequenced with an automated sequencer (ABI PRISM 3100 Genetic Analyzer, Applied Biosystems, Madrid, Spain) using a BigDye Terminator v3.1 Cycle Sequencing Kit (Applied Biosystems) according to the specifications provided by the manufacturer. The primers used for sequencing were the same as those used for PCR. The sequences were compared with the NCBI entries of *MC2R* (NM_000529), *MRAP* (NW_927384) and *StAR* (NG_011827.1).

### *In vitro* functional studies

Vectors expressing cDNAs of wild-type (wt) StAR and the fusion protein P450 side-chain cleavage/adrenodoxin/adrenodoxin reductase (F2) were a kind gift of Walter L. Miller, UCSF, San Francisco [1], [13]. Vectors expressing mutant StAR cDNA were generated by PCR-based, site-directed mutagenesis and confirmed by direct sequencing. Nonsteroidogenic COS-1 cells (CRL-1650™; http://www.atcc.org) were cultivated in DMEM (Invitrogen, Basel, Switzerland) supplemented with 10% fetal calf serum and antibiotics at 37°C. Cells were divided onto 12-well plates and grown overnight before transfection. Transfection with wt or mutant (T44H_S46X, G221S) StAR expression vectors in combination with F2 was performed with Lipofectamin 2000 (Invitrogen, Basel, Switzerland) in growth medium without antibiotics overnight. Transfected cells were then cultivated for another 24 hours. For assessing pregnenolone (Preg) production, cells were washed with phosphate buffered saline (PBS) and placed in DMEM without supplements; small aliquotes of supernatants were then collected over a time range of 5–600 min. Vector without StAR cDNA served as negative background control and the StAR-independent substrate 22R-hydroxycholesterol (22R OH-Chol) (Sigma-Aldrich, Buchs, Switzerland) added to the cell culture medium provided a positive control. Pregnenolone was determined by a commercially available ELISA (Labor Diagnostika Nord GmbH & Co, KG, Nordhorn, Germany). Sensitivity of this assay is 5 ng/dl. Data represent the mean ± SEM of 2 independent experiments performed in duplicates. Accuracy of this ELISA for pregnenolone measurement from culture medium was tested against pregnenolone measurements performed by GC/MS [14].

### Western blot

Fourty-eight hours after transfection, supernatants were collected and cells were washed with PBS and lysed. Protein content of the cell lysates was determined by the Bradford method (Biorad, Reinach, Switzerland). Protein lysates were separated on a 10% SDS-PAGE gel and transferred on a polyvinylidene difluoride membrane. Membrane was then incubated with a primary StAR antibody at a 1∶10′000 dilution (kindly provided by Walter L. Miller, San Francisco) and then with a goat-anti-rabbit secondary antibody at 1∶5′000 conjugated to HRP (Santa Cruz, Santa Cruz, CA, USA). The HRP signal was detected by an enhanced chemiluminescence staining (ECL, Amersham) and exposure to Amersham Hyperfilm MP (Amersham, GE Healthcare, Buckinghamshire, UK).

### Structural modeling of StAR

#### 3D protein models of N-62 StAR

The 3D structural model of StAR (**NP_000340.2,** P49675) sequence (62–285 AA) was based on the structures of StAR related lipid transfer domain of MLN64 [15] and human StAR related lipid transfer protein 5 (**1EM2-A** and **2R55-A**) that share high sequence similarity with human StAR proteins as obtained by 5 rounds of PHI BLAST iterated search of protein structure database. We performed several structural alignment with both template protein sequences that were then used for model building with the programs YASARA [16] and WHATIF [17]. First a secondary structure prediction of the StAR amino acid sequence was performed for correcting the alignment and for building the loops in the modeled structures with the program DSC [18]. A total of 10 different alignments (5 with each protein) were made and used for building ten different models. After building side chains, each model was subjected to first a steepest and then simulated annealing minimization for carrying out small refinement of the models. During this stage all backbone atoms of the aligned residues were kept fixed and only the sidechains were allowed to move. The corrected models were then subjected to a 100 ps simulated annealing energy minimization and then checked by the programs WHATCHECK [19], WHATIF [17], Verify3D [20], [21], ERRAT [22] and Ramachandran plot analysis [23], [24]. Coordinates of two template crystal structures (PDB# 1EM2 and 2R55) were used for comparative studies. After looking at the quality control results a hybrid model incorporating best quality models was made and refined by a 500 ps MD simulation. The MD simulations were performed using YASARA dynamics using AMBER03 force field [16]. In brief the cell was filled with water and the AMBER03 [25] electrostatic potentials was evaluated at all water molecules and ones with the lowest or highest potential were turned into a sodium or chloride counter ion until the cell was neutral. We then ran MD simulations with AMBER03 force field at 298 K and 0.9% NaCl in the simulation cell for 500 ps to refine the final model. Simulation trajectories were analyzed with WHATIF functions and snapshots of simulation were captured every 25 ps for further analysis. Best models were selected for analysis and evaluation of mutant amino acids. Computational site directed mutagenesis was performed using side chain optimizations by simulated annealing simulations energy minimizations and MD simulations over 500 ps. Structure models were depicted with Pymol (www.pymol.org) and rendered as ray traced images with POVRAY (www.povray.org).

### Steered MD simulation to study release of bound cholesterol by StAR

Final StAR-cholesterol complex obtained after docking and MD refinement was used to study the release of cholesterol by steered MD simulations. The cholesterol molecule was extracted from the StAR cavity by applying a constant force on the cholesterol molecule to drive it away from StAR during MD simulations. In brief, a force was applied on the center of cholesterol molecule to pull it away. Simulation was conducted with pH 4.0 to mimic the binding environment of StAR used in biochemical experiments and a distance range of 7.5–35Å. Experiment was repeated with S221-StAR.

## Results

### Identification of two novel StAR [c.125_126insG (p.Thr44HisfsX3) and c.661G>A (p.Gly221Ser)] mutations

**Figure 1:**
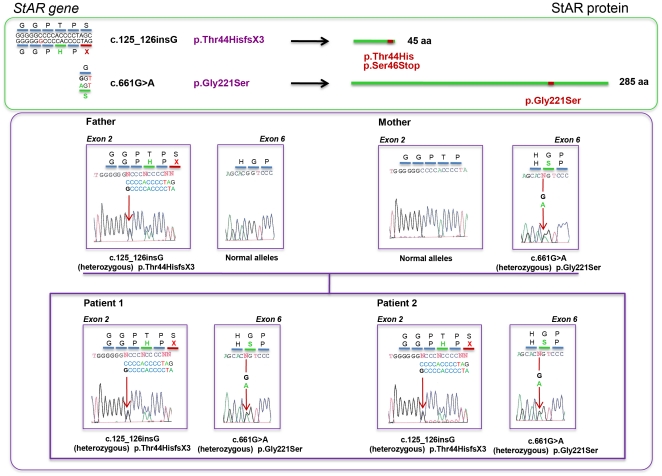
Genetic analysis of the StAR gene. Upper panel, scheme of the identified mutations at the nucleotide (c.DNA) and protein (p.) level. Lower panel, family tree with individual electropherograms showing the novel StAR mutations.

Two siblings initially suspected as having FGD, in whom *MC2R* and *MRAP* sequences were normal, were compound heterozygote carriers of two novel mutations in the *StAR* gene. A novel frameshift mutation in exon 2, c.125_126insG predicting p.Thr44HisfsX3 or T44H_S46X and a novel point mutation in exon 6, c.661G>A predicting p.Gly221Ser were identified (Figure 1). Analysis of the parents demonstrated that the exon 2 mutation was carried by the father while the exon 6 mutation was present on one allele in the mother (Figure 1).

### Structural model of StAR

**Figure 2:**
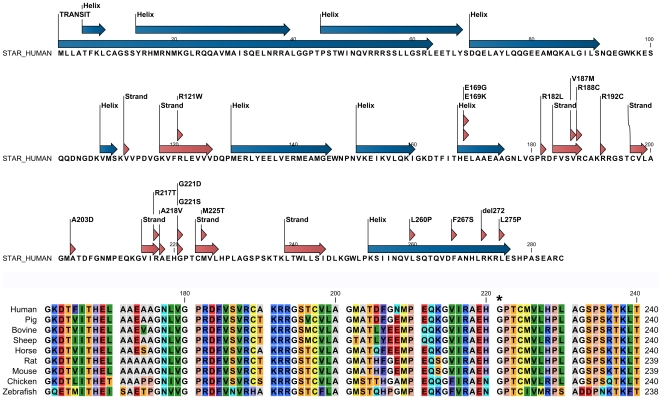
Secondary structural and amino acid conservation of human StAR. (A) A secondary structure prediction was performed to start the model building of human StAR. Locations of amino acids variants of StAR found in UniProt database and pubmed are indicated. (B) A partial sequencing alignment of human StAR amino acid sequence with a range of StAR proteins from other species found in the UniProt database. Overall, StAR amino acid sequence is very well conserved across species with only occasional difference showing up across whole alignment. All major residues at cholesterol binding pocket, E169, R188, R192 and T223 are conserved in all species studied in this analysis, while H220 was found to be replaced with an asparagine in Chicken and Zebrafish, potentially conserving the core structural elements.

**Figure 3:**
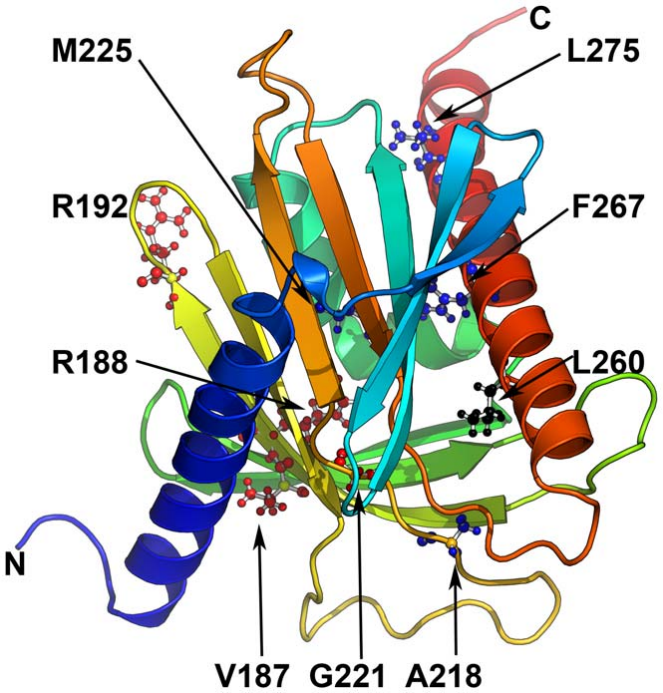
Model of StAR protein showing reported, non-classic StAR mutations. Mutations manifesting clinically with NCLAH and having >10% WT StAR activity in a direct functional assay (V187M, R188C, R192C, G221S) are depicted in red. Mutations A218V, M225T, F267S, L275P, for which clinical and functional results are conflicting, are given in blue. StAR loss-of-function mutation L260P is given in black for comparison.

We performed a PhiBlast search of PDB database with the amino acid sequence of human StAR. A custom position specific scoring matrix (PSSM) was created based on initial runs of the Phi-BLAST and after five rounds of search optimizations, the human StAR related lipid transfer domain of MLN64 (PDB # **1EM2**) and StAR related lipid transfer 5 (PDB # 2R55) structures were chosen as template based on structure quality and coverage of human StAR sequence. A secondary structure prediction of the human StAR sequence was performed to start the initial alignment (Figure 2). Amino acids 62 to 285 of the human StAR molecule were aligned with template structures to generate structural alignments of two molecules and fed into the YASARA and WHATIF programs for building of models. Some of the loop sequences were modelled separately by scanning loop database library. In the final model of StAR a total of 6 loops were modelled (LLGSRLEETL, QQDNGD, RMEAMG, VPDV, GSP, HPASEARC). Quality control analysis revealed 98.7% of residues in favoured regions and 100% residues in the allowed regions of the Ramachandran plot. Our StAR model was close to MLN64 in terms of core structural features and was also in agreement with previously described models of StAR (Figure 3) [26], [27], [28]. These previous models of StAR have also been based on MLN64 structure which was used as a template in our model building, and therefore, based on reported features, our model was quite similar in overall fold and geometry as compared to previous reports, and had similar steroid binding pocket, preserving core salt bridges between E169 and R188 [26], [27]. Changes in refinement protocols like use of newer amber2003 forcefield in our work compared to older amber99 forcefield used by Yaworsky et al [28], [29] may have resulted in minor changes in the modelling of side chains and inter atomic constraints. However, due to exponential increase in computational power available, we were able to use newer generation refinement algorithms, resulting in a final model that is of similar quality to a high resolution crystal structure. Another main difference in our model building have been the use of information in PDB_REDO database (http://swift.cmbi.ru.nl/gv/pdbredo/index.html) which re-refines the structures in PDB database using latest methods using the original structural data deposited in PDB, which tends to be several years old, and corrects the errors in structures found in PDB database [30], [31]. For the MLN64 structure (PDB # 1EM2), the overall Ramachanran plot appearance changed from −0.543 to 0.433 for the optimized entry, and total number of bumps/structural clashes were reduced from 61 in the original entry to 22, Chi-1/Chi-2 rotamer normality improved from −2.029 to 0.106, and R-free value changed from 0.2640 to 0.2115 in the optimized version used by us.

### Structural changes caused by the G221S mutation

**Figure 4:**
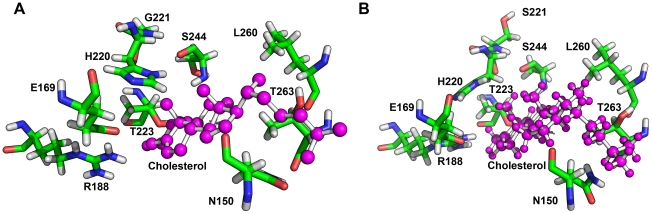
A closeup of the cholesterol binding pocket of StAR. After docking of cholesterol to both WT (A) and S221-StAR (B), we calculated potential residues interacting with cholesterol molecule during docking as well as exit of cholesterol from the binding cavity. In case of S221-StAR a loss of interaction with H220 was observed due to a shift in the H220 side chain. Cholesterol is shown as a ball and stick model in magenta. Amino acids interacting with cholesterol are shown as stick models.

To study the effect of the novel and some known StAR mutations, we performed computational mutagenesis of our structural model of StAR. To explore the role of mutations in binding and release of cholesterol by StAR, a docking of cholesterol into the StAR structure was performed and used to calculate the interactions with neighbouring amino acids forming the cholesterol binding pocket. Several hydrogen bonds with the hydroxyl group of cholesterol and side chains of amino acids forming the cavity holding the cholesterol seem to form during entry and exit of cholesterol. In our simulation, E169, R188, H220, T223 and S244 located on the beta sheets of the cholesterol binding cavity next to helix 3 were found to be involved in the interactions with cholesterol (Figure 4A). In addition, other potential interaction partners of cholesterol were N150 L260 and T263. In previous studies mutations of E169, R188 and L260 (E168 and R187 in case of hamster StAR) have been reported to result in loss of cholesterol transfer activity of StAR [2], [3], [5], [26]. Among these core residues, R188 forms salt bridges with E169 and H220, and two hydrogen bonds with E169, one of which was not found in G221S-StAR, although all salt bridges were intact. A slight but significant shift in the core binding site upon G221S mutation was observed due to disruption of G221–S244 hydrogen bond which seems to cause change in conformation of H220 side chain and disrupt interaction of H220 with cholesterol in the S221-StAR compared to WT-StAR as revealed by all atom contact analysis (Figure 4B). This change is likely to have resulted from additional contacts of S221 with V116 which are absent in WT StAR.

### Entry and exit of cholesterol in StAR

**Figure 5:**
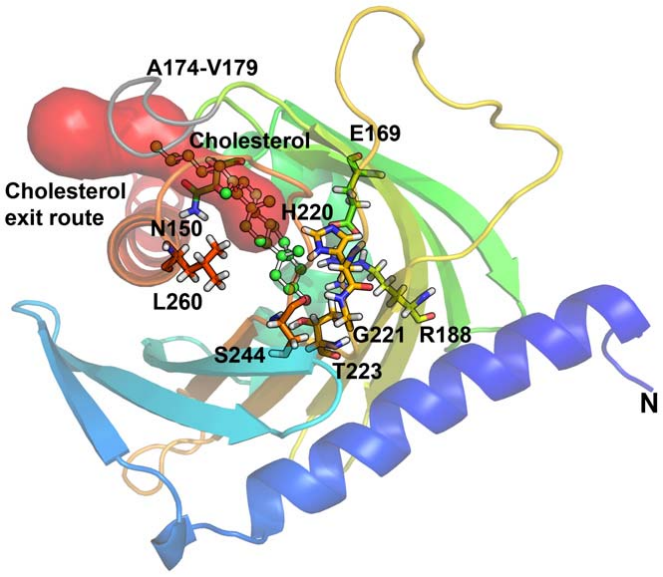
Exit of cholesterol from StAR as studied by steered MD simulations. StAR protein is shown as a ribbons model colored in a rainbow gradient from the amino terminus in blue to carboxy terminus in red. The A174–V179 loop that was observed to move and make way for the exit of cholesterol is shown in grey. Major amino acids involved in interaction of cholesterol with StAR and formation of cholesterol binding pocket are shown as stick models. The exit route of cholesterol observed during simulation is shown as a solid model in red. In case of S221-StAR a delay in exit of cholesterol was observed, potentially due to altered binding pattern caused by shift in H220 side chain and additional interactions with R188.

Potential routes of cholesterol entry and exit were also evaluated to fully understand the role of StAR mutation (G221S) described in this study. After docking of cholesterol into StAR, we performed a steered molecular dynamic simulation, where a constant force on cholesterol molecule was applied during simulation. We observed considerable movement of the loop formed by residues A174–L178 of StAR, but not of the C-terminus helix during the exit of cholesterol bound to StAR. An earlier report using the forced disulfide bond formation has indicated flexibility of C-terminus helix based on cholesterol binding studies with WT and structurally constrained StAR proteins [29]. Another recent computational molecular dynamic simulation study on binding and exit of cholesterol from StAR found movement of the A174–L178 loop but not the C-terminus helix [27], suggesting a possible rigid nature of the C-terminus helix. However, structures of both WT and structurally constrained StAR proteins reported by Baker et al [29] need to be solved to resolve the issue whether loss of cholesterol binding was due to putting constraints on C-terminus helix or by the subtle structural changes caused by the restraints created by artificially introduced disulfide bonds as suggested by Murcia et al [27]. Additional studies involving mutagenesis of amino acids in the loop formed by AA174–178 of StAR will also be required to investigate the potential mechanisms of cholesterol binding. We also observed interaction of cholesterol with R188 and T223 in WT and R188, T223 and S221 in G221S-StAR (Figure 5). Steered molecular dynamic simulations showed a slight but consistent delay of 2 ps in the release of cholesterol from the S221-StAR compared to WT StAR (10 versus 8 ps). Further analysis of cholesterol binding by looking at all the contacts revealed additional contacts between C5 and C6 of cholesterol molecule and R188 in case of S221-StAR that may be responsible for this delay. Therefore, potential mechanism for the partial loss of function of G221S-StAR could be due to the alterations in binding/release of the cholesterol molecule resulting from structural changes in the cholesterol binding pocket. It is possible that multiple modes of cholesterol binding and exit may exist in the function of StAR protein as suggested by two different models [27], [29] and any impairments caused by mutations may affect one or both of the mechanisms of cholesterol binding and exit in the StAR protein.

### Functional analysis of the novel StAR mutants *in vitro*

**Figure 6:**
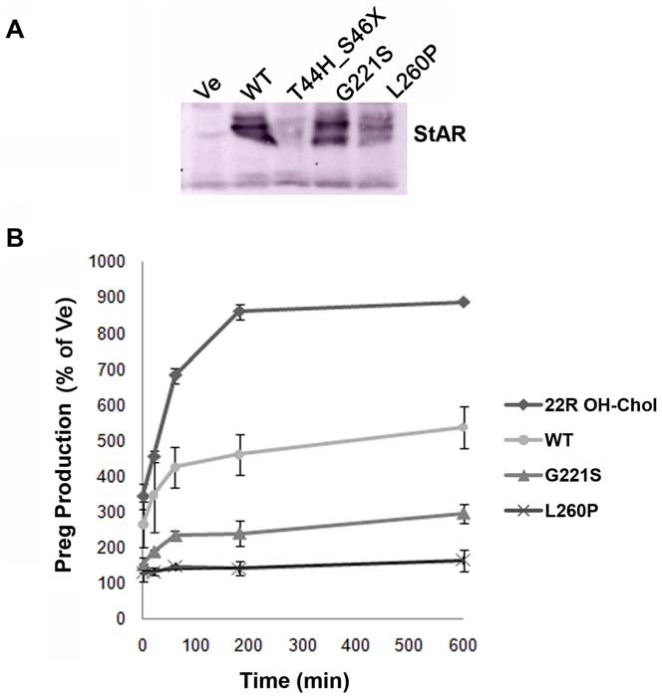
Functional testing of the identified StAR mutations *in vitro.* COS-1 cells were transiently transfected with expression vectors for the side chain cleavage system (F2) and wild-type (WT) or mutant StAR. A) Protein expression was assessed by Western blot. B) The ability to produce pregnenolone (Preg) was measured in the cell supernatants after 5–600 min using a commercially available ELISA assay. Results are given as mean ± SD. StAR independent Preg production of the cell system was assessed by using empty vector/F2 and adding 22(R)-hydroxycholesterol (22R OH-Chol) to the cell medium.

To assess the impact of the newly identified StAR mutations on steroidogenesis, we generated expression vectors and compared their ability to promote pregnenolone production to wt StAR in an established cellular model [1]. Western blot analysis confirmed similar levels of expression of both the mutant G221S and the wt StAR protein (Figure 6A). By contrast, the StAR mutation T44H_S46X which is predicted to shorten the StAR protein from 285 amino acids to only 45 amino acids, was not detected in our Western blots, either due to degradation of smaller protein fragment or smaller fragment running off the gel. Assessment of pregnenolone production in cell supernatants by ELISA revealed similar pregnenolone production rate for the T44H_S46X mutant as for vector control (data not shown on the graph). By contrast, compared to the wt StAR the G221S mutant produced 30–50% pregnenolone over the observation period of 5–600 min while the L260P mutant (which is associated with severe CLAH [32]) produced pregnenolone only to the basal levels of the vector control (Figure 6B).

## Discussion

Two siblings (a boy and a girl) were diagnosed 30 years ago with congenital adrenal hyperplasia and were treated with glucocorticoids and mineralocorticoids although no clear signs of salt loss were present except for a moderately elevated PRA in the girl. Mineralocorticoid therapy was withdrawn in the boy at the age of 17 years without clinical problems as expected, while the sister continues with the therapy due to moderately low plasma sodium values with normal potassium levels, although the PRA has not been assessed further. These familial cases were considered to have a FGD syndrome and analyzed for the candidate genes. After finding that *MC2R* and *MRAP* gene sequences were normal, two novel *StAR* mutations were identified, one of paternal, the other of maternal origin. This raises the number of families with reported late-onset, nonclassic CLAH (NCLAH) with *StAR* mutations to over 10 (Table 1).

**Table 1:** Missense mutations identified in patients manifesting with non-classic StAR deficiency.

Mutation	H = homozygote;C = compound heterozygote*	Number of families	Karyotype in reported patients	Age at manifestation of clinical signs (A = adrenal, S = sexual development/reproduction)		*In vitro* activity (% of wild-type)***	Ref.
				A	S		
V187M	H	1	XX	4.5 yrs	–	22	3
R188C	H and C	7	XX and XY	1–58 yrs	Variable at birth with none in XX or cryptorchidism and mild hypospadias in few XY; some fertility issues in XY adults	8–14	3, 4, 5
R192C	H	1	XX and XY	5 yrs	Hypofertility at 25 yrs (XY)	40	4, 5
A218V	C (p.Q258X)	2	XX and XY	2 wks	Severe 46,XY DSD	14	36
G221D	H	1	XX	11 mo	–	3	5
G221S	C (p.T44H_S46X)	1	XX and XY	10 mo	–	30–50	This study
M225T	C (p.Q258X)	1	XY	10 mo	Severe 46,XY DSD	43	36
F267S	C (p.L260P)	1	XY	10 wks	Severe 46,XY DSD	6	5
L275P	C (p.A218V)	1	XY	2 mo	Severe 46,XY DSD	24	2
*L260P* **	*H*	*4*	*XX and XY*	*At birth*	*46,XY DSD*	*3*–*10*	5, 32

*Mutation on partner allele is given in brackets (p.).

**Loss of function mutation manifesting clinically at birth with signs of classic StAR deficiency. Data given for comparison.

***In vitro activity (% of WT) is assessed by pregnenolone production (immunoassay) in COS cells transfected with expression vectors for wild-type or mutant StAR and F2 (the fusion protein P450 side-chain cleavage/adrenodoxin/adrenodoxin reductase). Note that data derive from different laboratories employing similar assay systems.

Like in other reported cases of NCLAH (Table 1), gonadal function was normal during fetal life in the boy and in both siblings during pubertal development and through adulthood up to their present age. The female patient has regular menses and normal gonadotropin levels at the age of 32 years; the male patient has normal serum testosterone with gonadotropin levels at the upper normal limit and inhibin B in the lower normal range at age 29 years. As neither of them has attempted to have children, fertility remains unanswered. Similarly, evolution of their gonadal function remains uncertain. The cases of (N)CLAH reported to date show a wide variation in the gonadal function involvement in both females and males. In 46,XX females, this may range from normal fertility and age at menopause to infertility, progressive increase in LH and development of ovarian cysts [2], [33], [34]. In 46,XY individuals sexual development may be severely affected or only minor anomalies may be found (hypospadias and/or cryptorchidism) or even absent at birth [1], [2], [3], [4], [5], [32], [35]. Accordingly, 46,XY individuals without major genital anomalies may have normal pubertal development and adulthood sexual function; however, infertility may also develop gradually with age [4]. The overall sex difference in gonadal failure, which is even seen with the loss-of-function StAR mutations, is thought to be explained by the fact that the ovaries are quiescent and inactive from fetal life to puberty but the testes are fully active in a 46,XY fetus [2], [33]. Therefore, as the stimulation of steroidogenesis in a StAR deficient milieu will lead to accumulation of cholesterol and cell damage over time [2], it seems plausible that partial loss of StAR will damage steroidogenic cells to a lesser degree, and that the adrenal cortex and the testes (both of which are fully active in utero) will be affected earlier than the ovaries.

The age at the presentation and the diagnosis of adrenal failure and the degree of mineralocorticoid deficiency show large variations among the patients described (Table 1), ranging from adrenal failure at 2–4 years of age with mild hyperreninemia in the first described patients [3] to the lack of diagnosis and treatment at 58 years of age without evidence of mineralocorticoid deficiency in another series [4]. Our patients presented with typical symptoms of adrenal failure (hypoglycemia and dehydration) at the age of 10 and 14 months; both had cortisol deficiency but no obvious mineralocorticoid deficiency.

To date 9 StAR mutations have been reported as causing NCLAH because of clinical manifestations and/or *in vitro* functional testing (Table 1). Recently, a comparison of three *in vitro* assays to test StAR function revealed a significant inter- and between assay variability with the best phenotype correlation coming from the direct assays measuring pregnenolone production [5]. It appeared that StAR mutations with more than 10–20% of wt activity *in vitro* may rather manifest as NCLAH (or FGD) while an activity of less than 10% may be found with severe forms of CLAH. Concordant clinical and functional findings suggested that NCLAH exists for StAR mutations V187M, R188C, R192C, and G221S [3], [4], [5]. Similarly, G221D StAR was manifesting with isolated cortisol deficiency, but revealed only 3% activity in the functional assays [5]. As revealed by our structural model of StAR, all these residues are at the cholesterol binding pocket with R188 making a direct contact with the cholesterol. A disruption in this interaction by mutation of the R188 residue or the structural shifts in the cholesterol binding pocket due to mutations of neighbouring residues will likely cause disruption in either the binding or the release of cholesterol. Although StAR mutations A218V, M225T and L275P have been tested in vitro as being partially active (14%, 43%, 24% respectively), clinical findings of patients harboring these mutations qualify for the classic form with neonatal onset of disease and/or severe 46,XY DSD at birth [2], [36]. The C-terminus helix in StAR provides the boundary for cholesterol binding pocket and both L260 and F267 are involved in interaction with cholesterol during binding/release. The F267S mutation had low in vitro activity, manifested with a severe form of 46,XY DSD and early adrenal insufficiency (10 weeks) [5]; and qualifies therefore for the classic form of CLAH.

In summary, we report two novel StAR mutations found in compound heterozygote form in two related patients (sister and brother) presenting with isolated glucocorticoid deficiency. The T44H_S46X mutation encoded a 45 aa protein which was completely inactive. The G221S StAR mutation retained more than 30% of wt activity explaining the mild phenotype.
